# Effect of FADS1 SNPs rs174546, rs174547 and rs174550 on blood fatty acid profiles and plasma free oxylipins

**DOI:** 10.3389/fnut.2024.1356986

**Published:** 2024-07-03

**Authors:** Miriam Rabehl, Zeren Wei, Can G. Leineweber, Jörg Enssle, Michael Rothe, Adelheid Jung, Christoph Schmöcker, Ulf Elbelt, Karsten H. Weylandt, Anne Pietzner

**Affiliations:** ^1^Medical Department B, Division of Hepatology, Gastroenterology, Oncology, Hematology, Palliative Care, Endocrinology and Diabetes, University Hospital Ruppin-Brandenburg, Brandenburg Medical School, Neuruppin, Germany; ^2^Faculty of Health Sciences, Joint Faculty of the Brandenburg University of Technology, Brandenburg Medical School and University of Potsdam, Potsdam, Germany; ^3^Brandenburg Institute for Clinical Ultrasound, Brandenburg Medical School, Neuruppin, Germany; ^4^Experimental Lipidology, Brandenburg Medical School, Neuruppin, Germany; ^5^Medical Department, Division of Psychosomatic Medicine, Campus Benjamin Franklin, Charité-Universitätsmedizin Berlin, Corporate Member of Freie Universität Berlin and Humboldt-Universität zu Berlin, Berlin, Germany; ^6^Lipidomix, Berlin, Germany

**Keywords:** steatosis hepatis, MAFLD, FADS1, Oxylipins, PUFA

## Abstract

**Introduction:**

Previous studies have indicated that activity of fatty acid desaturase 1 (FADS1), is involved in cardiometabolic risk. Recent experimental data have shown that FADS1 knockdown can promote lipid accumulation and lipid droplet formation in liver cells. In this study, we aimed to characterize whether different FADS1 genotypes affect liver fat content, essential fatty acid content and free oxylipin mediators in the blood.

**Methods:**

We analyzed the impact of FADS1 single-nucleotide polymorphisms (SNPs) rs174546, rs174547, and rs174550 on blood fatty acids and free oxylipins in a cohort of 85 patients from an academic metabolic medicine outpatient center. Patients were grouped based on their genotype into the homozygous major (derived) allele group, the heterozygous allele group, and the homozygous minor (ancestral) allele group. Omega-3 polyunsaturated fatty acids (n-3 PUFA) and omega-6 polyunsaturated fatty acids (n-6 PUFA) in the blood cell and plasma samples were analyzed by gas chromatography. Free Oxylipins in plasma samples were analyzed using HPLC–MS/MS. Liver fat content and fibrosis were evaluated using Fibroscan technology.

**Results:**

Patients with the homozygous ancestral (minor) FADS1 genotype exhibited significantly lower blood levels of the n-6 PUFA arachidonic acid (AA), but no significant differences in the n-3 PUFAs eicosapentaenoic acid (EPA) and docosahexaenoic acid (DHA). There were no significant differences in liver fat content or arachidonic acid-derived lipid mediators, such as thromboxane B2 (TXB2), although there was a trend toward lower levels in the homozygous ancestral genotype group.

**Discussion:**

Our findings suggest that FADS1 genotypes influence the blood levels of n-6 PUFAs, while not significantly affecting the n-3 PUFAs EPA and DHA. The lack of significant differences in liver fat content and arachidonic acid-derived lipid mediators suggests that the genotype-related variations in fatty acid levels may not directly translate to differences in liver fat or inflammatory lipid mediators in this cohort. However, the trend towards lower levels of certain lipid mediators in the homozygous ancestral genotype group warrants further investigation to elucidate the underlying mechanisms of different FADS1 genotypes and potential implications for cardiometabolic risk.

## 1 Introduction

Metabolic-dysfunction associated fatty liver disease (MAFLD, steatosis hepatis) is a common disease with an estimated prevalence of 20–30% (1, 2). It is the most common liver disease in the Western world, with a rising trend. MAFLD can be considered as hepatic manifestation of the metabolic syndrome (MetS) (3, 4). MAFLD patients are at risk to develop persistent inflammation (Nonalcoholic steatohepatitis, NASH), which can lead to liver fibrosis, cirrhosis and is associated with an increased risk of developing hepatocellular carcinoma (5, 6).

Genes involved in the elongation and desaturation of long-chain (lc) PUFAs from short-chain precursors have been linked to diet-dependent risks of cardiovascular disease (CVD) and metabolic syndrome MetS) (7–9).

**Figure 1:**
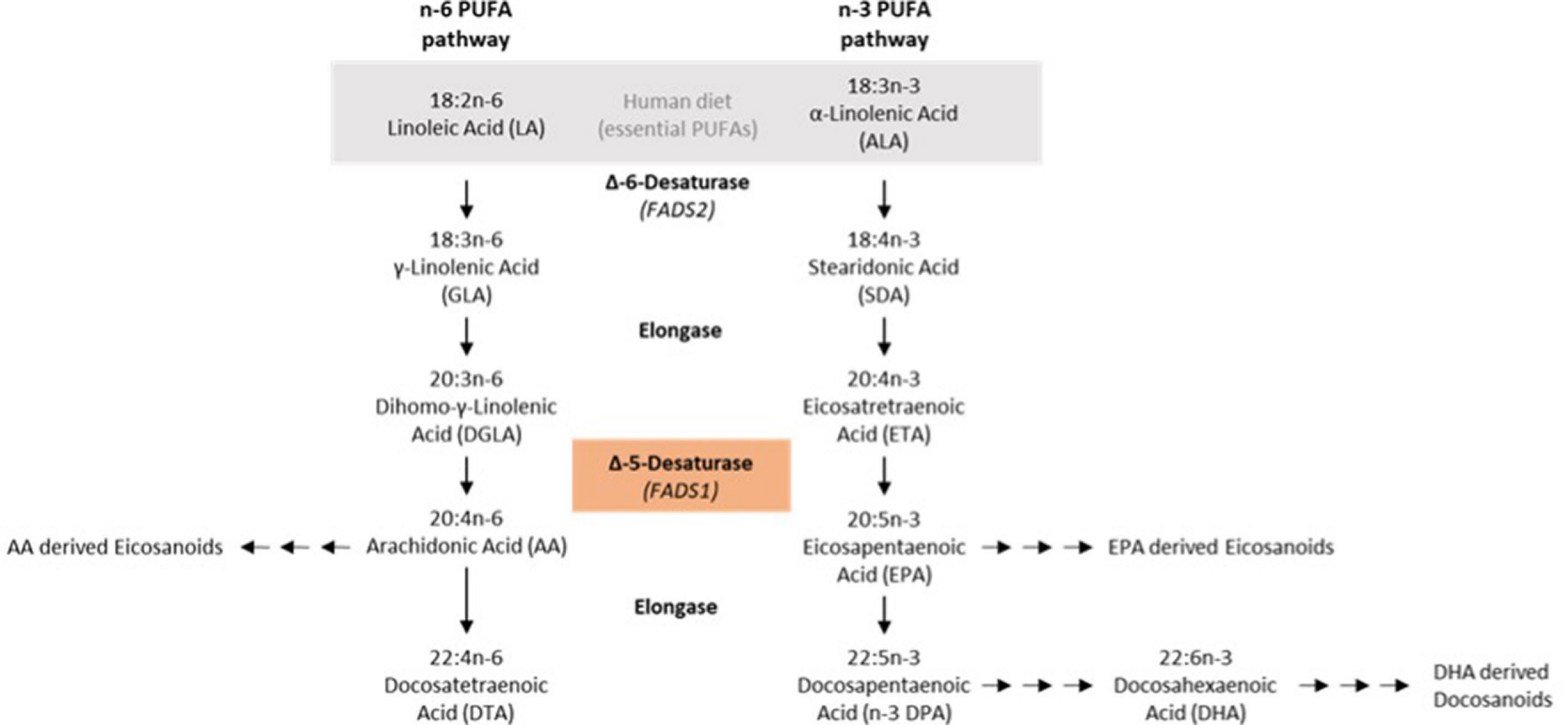
N-6/n-3 PUFA synthesis pathways.

One of the rate-limiting steps in the synthesis of lc-PUFAs from sc-PUFAs is catalyzed by the fatty acid (FA) delta-5-desaturase (D5D; Figure 1) (10). D5D or fatty acid desaturase 1 (FADS1) is a membrane-bound desaturase that catalyzes the synthesis of lc omega-3 (n-3) and omega-6 (n-6) PUFAs from dietary linoleic acid (LA, 18:2 n-6) and α-linolenic acid (18:3 n-3) (11). The human D5D gene is located on chromosome 11 in the *FADS* region, where several polymorphisms are located. These *FADS* gene polymorphisms affect PUFA synthesis in addition to dietary regulation of FA supply and composition (10). Two alleles of the SNPs rs174546, rs 174547 and rs174550 are distinguished at the *FADS1* locus. The major or derived allele corresponds to the following bases at these three SNPs: C at rs174546, T at rs 174547 and T at rs174550. The minor or ancestral allele corresponds to the following bases at the three SNPs analyzed here: T at rs174546, C at rs174547 and C at rs174550. The minor alleles of these SNPs in the FADS1 gene were found to be associated with lower blood concentrations of lc n–3 and n–6 PUFAs (12–14). Some data suggest that the major alleles are associated with an increased risk of atherosclerosis in the context of a diet high in animal fats (15), whereas other studies found that the minor alleles might confer an increased cardiovascular risk that could be alleviated by dietary measures (16).

It has been shown that the *FADS1* minor ancestral allele has been the only relevant allele in human populations in Europe until approximately 8.500 years ago, when the derived allele was introduced (17).

The minor ancestral allele, which is characterized by lower *FADS1* expression, has been implicated to confer increased metabolic risk and increased liver steatosis (18–21).

In this pilot study, we aimed to characterize liver fat content and lipidomics characteristics from patients enrolled in a routine setting of an outpatient metabolic medicine center for diagnosis, monitoring or treatment of MetS and MAFLD. We wanted to determine whether the homozygous minor (ancestral) FADS1 genotype is associated with higher liver fat in unselected patients presenting to our center, and whether different FADS1 genotypes affect essential fatty acid content and free oxylipin mediators in the blood.

## 2 Methods

Patients presenting to our metabolic disease clinic were recruited, containing patients with risk for, or manifest MAFLD, MetS, type 2 diabetes mellitus (T2DM), and/or dyslipidemia (elevation of triglycerides, total cholesterol, or LDL cholesterol or lowering of HDL cholesterol). Patients gave their informed consent for this research project investigating essential n-6 and n-3 fatty acids in the context of metabolic disease (approved by the institutional ethics committee, Nr. Z02-20170508). Only patients with signed informed consent were included in the study.

To assess PUFA levels in blood cells and plasma, blood samples were collected after at least 6 h of fasting from patients (*n* = 85) enrolled in a routine setting. All samples were centrifuged at 3500 rpm for 10 min at 4°C, separated blood cell and plasma samples were stored at −80°C until FA analysis. Extraction and quantification were carried out according to established protocols. According to a previous study, we expected a prevalence of patients homozygous for the minor FADS1 allele (that has lower D5D activity) of 11% (16), and aimed for 9–10 patients with this genotype to allow for meaningful statistical analysis of the FA and oxylipins.

In brief, FA from 50 μL erythrocytes and 75 μL plasma per sample were analyzed. Boron trifluoride (BF_3_) derivatization was applied for the blood cell fraction (22) and a combined BF_3_ + NaOH method for derivatization was used for the plasma samples (23, 24). FA values from blood cells are presented as percentage [%] of total FA content, FA concentration from plasma are presented in absolute amounts [μg/ml]. Free Oxylipins in plasma samples [ng/ml] were analyzed as described previously using LC–MS/MS (Lipidomix, Berlin) (25).

Sonographic evaluation was performed using FibroScan (Echosens, Paris), a standardized and reproducible point-of-care technique for quantification of steatosis and fibrosis as described previously (25). This is a non-invasive measurement method for quantifying a fatty liver, in which acoustically generated transient ultrasound waves cause the liver to vibrate intermittently. This method is based on shear wave elastography (SWE) (26).The cutoff value reported in the literature for the detection of hepatic steatosis ranges from 222 decibels per meter (dB/m) in a cohort of patients with chronic hepatitis C to 294 dB/m in a meta-analysis of patients with nonalcoholic fatty liver disease (NAFLD) (27). The examination was performed in all patients in a fasting state (the last meal should have been at least 6 h before the measurement). The examination was performed in the supine position with maximal abduction of the right arm and positioning of the right leg over the left to gain sufficient intercostal access. The probe was placed at the intersection of the xiphoid process and the midaxillary line. Patients weighing <100 kg were examined with the M probe (standard probe—transducer frequency 3.5 MHz) and patients weighing >100 kg were examined with the XL probe (transducer frequency 2.5 MHz). Reliable measurements were defined as: Median of 13 valid LS measurements with an interquartile range ≤ 30% (IQR/med = the difference between the 75 and 25th percentiles, i.e., the range of the middle 50% of the data relative to the median).

The *FADS1* SNPs rs174546, rs174547 and rs174550 were characterized in venous blood collected from the patients in EDTA tubes using an Illumina Platform (Life and Brain, Bonn, Germany).

For statistical analysis FA or oxylipin values were tested for normal distribution using the Shapiro–Wilk test. For normally distributed values, a one-way ANOVA between the three groups and Tukey’s Honest Significance Difference (HSD) test as follow-up to assess significances between subsets of two groups was performed. For non-normally distributed values, testing for significant differences between the three groups was performed using the Kruskal-Wallis test and followed up with Dunn’s testing for subsets of the possible pairwise comparisons. Categorical values (age) were compared using Pearson Chi Square testing. Statistical analyses were done using GraphPad prism or Excel software. *p* ≤ 0.05 was considered as significant.

## 3 Results

We aimed to include at least 10 patients homozygous for the minor (ancestral) *FADS1* allele. This objective was achieved after having screened 85 patients for whom we performed liver fat determination, FA analyses and, of a subset, lipidomics analyses. Out of these 85 patients, 37 were homozygous for the major (derived) *FADS1* alleles (rs174546 CC, rs174547 TT and rs174550 TT), 37 heterozygous (rs174546 CT, rs174547 CT and rs174550 CT), and 11 homozygous for the minor (ancestral) *FADS1* allele (rs174546 TT, rs174547 CC and rs174550 CC). As shown in Table 1 there were no significant differences between the groups regarding age and sex. Furthermore, there were no significant differences between groups regarding steatosis and fibrosis, as assessed by Fibroscan.

**Table 1:** Liver fat and fibrosis parameters of included patients depending on rs174546 genotype.

	Total	CC	CT	TT
	*n* = 85	*n* = 37	*n* = 37	*n* = 11
N (Female/Male)	36/49	11/26	19/18	6/5
Age (years)	52.8 ± 1.7	53.2 ± 2.6	54.2 ± 2.6	46.6 ± 4.9
CAP (dB/m)	283.0 ± 7.1	290.4 ± 10.5	281.6 ± 10.8	267.3 ± 20.6
LSM (kPa)	10.4 ± 1.6	11.0 ± 2.6	8.8 ± 2.0	13.5 ± 5.1

Data are given as data as mean ± SEM, CAP, common attenuation parameter in dB/m (decibels per meter), LSM, liver stiffness measurement in kPa (kilopascal). All differences between groups were not significant assuming a *p*-value of < 0.05 for significance.

In order to assess FADS1 activity the delta-5-desaturase index (D5D index) was calculated as the following ratio of arachidonic acid (AA) and its precursor dihomo-gamma-linolenic acid (DGLA) in FA and plasma (10, 28):


$$D5D\;\mathrm{index}=\;\frac{\mathrm{arachidonic}\;\mathrm{acid}\;\left(\mathrm{AA};C20:4n-6\right)}{\mathrm{dihomo}-\mathrm{gamma}-\mathrm{linoleinic}\;\mathrm{acid}\;\left(\mathrm{DGLA};C20:3n-6\right)}$$

**Figure 2:**
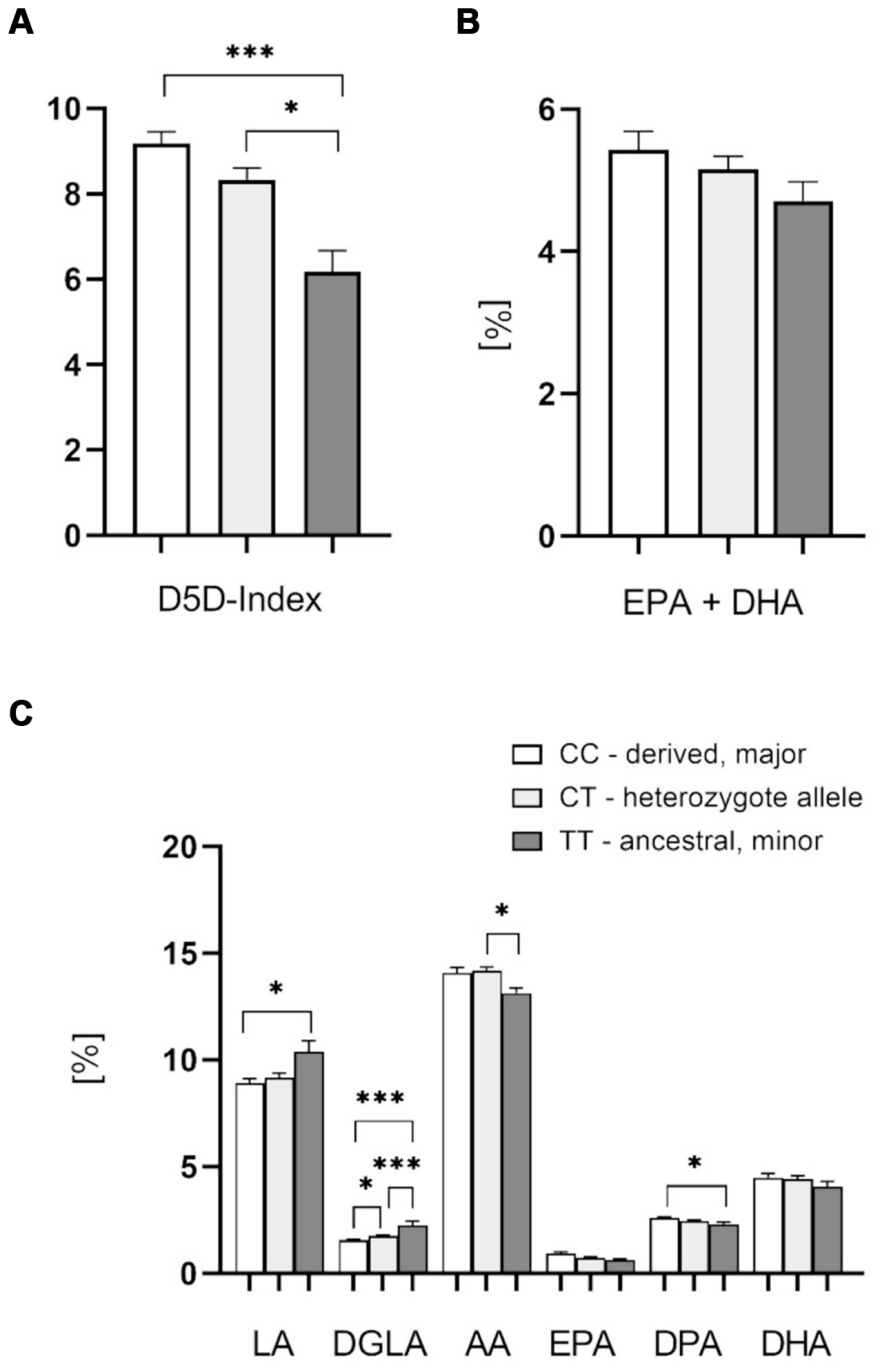
Effect of the FADS1 rs174546 genotype on PUFAs in the blood cell fraction. **(A)** D5D index is significantly higher with the C allele carriers (CC > CT > TT). **(B)** Slightly lower EPA + DHA content (determined in analogy to the Omega-3-Index) in the blood cell fraction from patients with the homozygous ancestral FADS1 genotype (TT). **(C)** Significantly higher levels of n-6 PUFAs linoleic acid (LA) and dihomo-gamma-linolenic acid (DGLA), and lower levels of arachidonic acid (AA) and n-3 PUFA docosapentaenoic acid (DPA) with the minor TT genotype. *n* = 37 for the homozygous derived genotype CC, *n* = 37 for the heterozygous genotype CT, *n* = 11 for the homozygous ancestral genotype TT, *p < 0.05, **p < 0.01, ***p < 0.001, one-way ANOVA with subsequent Tukey’s HSD testing.

As expected from previously published data we found highly significant D5D index differences between genotypes containing the major allele (rs174546 CC and CT,) and the homozygous minor allele (rs174546 TT), supporting higher FADS1 gene activity with the major derived C allele, leading to higher levels of AA and lower levels of DGLA (Figures 2A,C). Indeed, there were significant differences in blood cell FA content for LA (18:2 n-6), DGLA (20:3 n-6), AA (20:4 n-6), and n-3 docosapentaenoic acid (DPA, 22:5 n-3). No significant differences were found for eicosapentaenoic acid (EPA, 20:5 n-3) and docosahexaenoic acid (DHA, 22:6 n-3; Figure 2C). The content of EPA plus DHA, determined in analogy to the well-established Omega-3-Index (29), was slightly lower in the homozygous minor allele group (Figure 2B).

**Figure 3:**
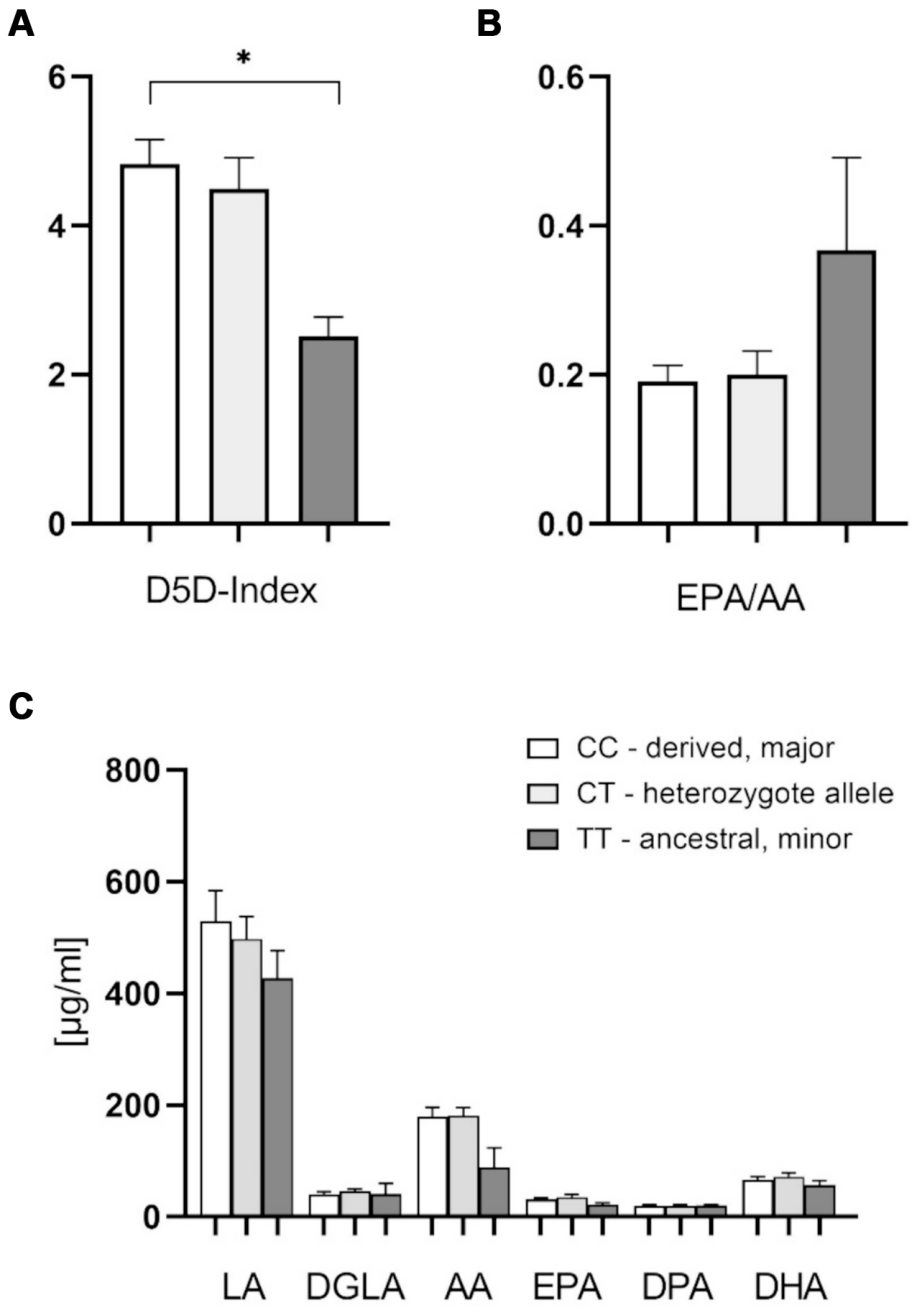
Effect of the FADS1 rs174546 genotype on plasma PUFAs content. **(A)** The D5D index is significantly higher with the CC alleles in comparison to TT alleles. **(B)** Slightly higher EPA/AA plasma ratio with the homozygous ancestral FADS1 genotype (ns, *p* = 0.067). **(C)** Slightly lower AA (20:4 n-6) levels with the homozygous ancestral FADS1 genotype. *n* = 19 for the homozygous derived genotype, *n* = 19 for the heterozygous genotype, *n* = 4 for the homozygous ancestral genotype, *p < 0.05, Kruskal-Wallis and subsequent Dunn’s testing.

In a subset of patients, we also analyzed plasma fatty acids. These data confirmed the significantly higher D5D index, as well as higher levels of AA (20:4 n-6) in the derived genotype carriers (Figures 3A,C). There was a significant difference of D5D indices between the homozygous CC and TT allele carriers. In this subset we did not find significant differences for other plasma FAs. Interestingly, there was a higher plasma EPA/AA-ratio in patients with the homozygous ancestral genotype, although this finding did not reach statistical significance (Figure 3B). This parameter, with a cutoff at 0.4 and higher levels being beneficial, has been implicated as a risk stratification marker for cardiovascular protection (30, 31).

**Figure 4:**
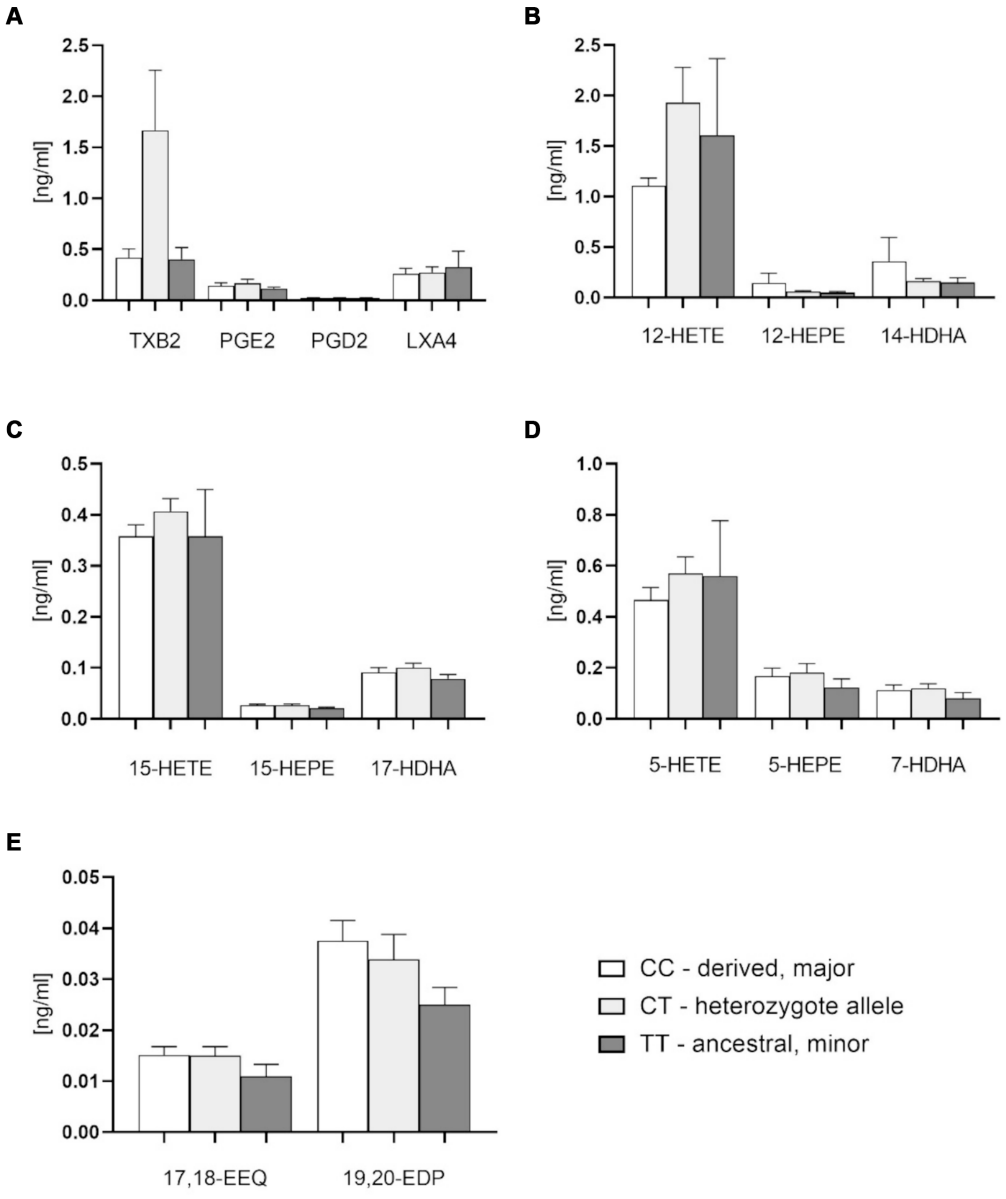
Plasma free oxylipins in groups with FADS1 rs174546 genotypes. **(A)** Thromboxane B2 (TXB2), prostaglandin D2 (PGD2), prostaglandin E2 (PGE2) and lipoxin A4 (LXA4) contents in plasma of different FADS1 genotypes. **(B)** 12-Lipoxygenase-derived oxylipin contents from AA (12-hydroxyeicosatetraenoic acid, 12-HETE), EPA (12-hydroxyeicosapentaenoic acid, 12-HEPE) and DHA (14-hydroxydocosahexaenoic acid, 14-HDHA) in plasma of different FADS1 genotypes. **(C)** 15-Lipoxygenase-derived oxylipin contents from AA (15-HETE), EPA (15-HEPE) and DHA (17-HDHA). **(D)** 5-Lipoxygenase-derived oxylipins from AA (5-HETE), EPA (5-HEPE) and DHA (7-HDHA) in plasma of different FADS1 genotypes. **(E)** Cytochrome P450-derived epoxy metabolites from EPA (17,18-epoxyeicosatetraenoic acid 17,18-EEQ) and DHA (19,20-epoxydocosapentaenoic acid, 19,20-EDP) in plasma of different FADS1 genotypes. *n* = 36 for the homozygous derived genotype, *n* = 36 for the heteozygous genotype, *n* = 10 for the homozygous ancestral genotype. There were no significant differences between groups from Kruskal-Wallis testing.

There were no significant differences of plasma free oxylipin content between different FADS1 genotypes, with slightly higher, but not significantly different levels of metabolites in the homozygous ancestral genotype as compared to the other genotypes (Figures 4A–E). There were indications, however, that platelet-related mediators such as thromboxane B2 (TXB2) and 12-HETE tended to be higher with the derived genotype (Figures 4A,B). Potentially hepatoprotective epoxy metabolites also showing a trend to higher levels as compared to the minor homozygotes (Figure 4E).

## 4 Discussion

The development of MetS is affected not only by lifestyle but also by genetics. In a retrospective study, the difference of dietary patterns was not enough to cause the change of MetS outcomes (32). Since genetic variants affect lipid metabolism which is closely associated with MetS (33), attention focused on the association between MetS and genetic factors (34–36), and FADS genotype has been found to contribute to biochemical and metabolic variations in the MetS. In our pilot study in a routine clinical setting, we chose the *FADS1* SNP rs174546 which has been reported to be in linkage disequilibrium (LD) with other *FADS1* variants as a representative variant for differences in the FADS1 genotype (12, 36) and confirmed this with the SNPs rs174547 and rs174550.

Carriers of the major allele are known to have higher FADS1 enzyme activities than homozygous carriers of the ancestral minor allele. This leads to higher substrate precursor concentrations (LA, 18:2 n-6), higher substrate concentrations (DGLA, 20:3 n-6) and lower product (AA, 20:4 n-6) concentrations in patients homozygous for the minor alleles (12, 16, 37).

We confirmed the previously described significantly lower D5D index and activity for patients with the homozygous minor (ancestral) *FADS1* allele as compared to those homo- or heterozygous for the major (derived) allele in the blood cell fraction as well as in plasma for homozygous minor allele carriers in comparison with homozygous major allele carriers. Furthermore, the amounts of LA (18:2 n-6) and DGLA (20:3 n-6) were significantly higher in blood cells with the homozygous ancestral FADS1 genotype, whereas amounts of AA (20:4 n-6) and DPA (22:5 n-3) were significantly lower.

The lc n-3 PUFA concentrations in plasma and blood cells also tended to show higher concentrations in the derived allele carriers. This also indicates increased D5D activities in the derived allele carriers. We could not find any significance here except for n-3 DPA contents in blood cells, with higher values in the derived allele carriers. The observed reduction in D5D product concentrations of n-3 LC PUFAs, compared to the significant differences in n-6 LC PUFAs among derived allele carriers, may result from an excess of available linoleic acid (LA).

Our findings regarding D5D index and n-6 PUFAs are in agreement with earlier studies. EPIC, a multicenter prospective cohort study analyzing 2,653 patients, the TT genotype at SNP rs174546 was inversely related to D5D activity (39). Another large study also observed that D5D activity was significantly lower among carriers of the rs174546 minor T allele. In addition, this study also indicated higher LA and DGLA concentrations in patients with the TT genotype, while lower concentrations were observed for AA (40). In the PREOBE cohort, women who carried the T allele at rs174546 had higher DGLA and lower AA levels (41). Furthermore, higher serum phospholipid concentrations of LA were observed in people with the T allele of rs174546, while the lower serum phospholipid levels were observed for AA (42).

However, contrary to earlier studies (15, 43–45), we did not observe that FADS1 gene variants were associated with significant differences in blood levels of n-3 PUFAs EPA or DHA. Interestingly, other data also indicate that genetic differences at the *FADS1* and *FADS2* loci probably have stronger effects on n-6 PUFAs than n-3 PUFAs (46). Having said that, these differences could also be due to differences in dietary consumption of n-3 PUFA, for which there is more variation than for n-6 PUFA in western diet between individuals with high or low fish consumption, which we did not monitor in our study.

Liver steatosis and fibrosis assessed by Fibroscan (47) were not associated with FADS1 allele variations, which is in contrast to a pediatric study indicating that the minor *FADS1* variant was associated with a higher degree of liver steatosis (19).

In order to expand knowledge and analyses regarding FADS1 genotype variations we also performed oxylipin analyses in plasma. While we did not observe significant differences between the groups, we found a trend toward higher thrombocyte AA-mediator formation in the derived group—which might indicate higher vascular risk and could fit with earlier observations that the derived FADS1 genotype is associated with increased cardiovascular risk (48, 49). Furthermore, we found a trend toward a lower plasma EPA/AA ratio in the derived group. At the same time n-3 PUFA derived epoxy metabolites that were identified as steatosis-protective factors in mouse experiments (50, 51), were lower in the ancestral group. This could be a mechanism involved in increased steatosis and metabolic risk that has been described for patients with the ancestral genotype (19, 21). Our data are not sufficient to prove these effects, however, they form the basis for hypotheses that now need to be tested in other studies.

Limiting factors of this observational study are mainly the heterogeneous group composed of a random selection of patients. In addition, the group of homozygous ancestral allele carriers is relatively small. In future studies it will be important to collect additional clinical data from patients, such as routine lipid parameters, blood pressure measurements and BMI, to further stratify risk constellation for steatosis hepatis and cardiovascular disease. Looking at the FADS1 gene alone is also a limitation. For example, Shetty et al. described an association of FADS2 gene polymorphisms with increased insulin resistance and type 2 diabetes (52). In the current cohort, diabetes mellitus status was not considered and thus the inclusion of this criterion is needed to better assess the risk constellation between diabetes mellitus and cardiovascular risk in MAFLD.

To further assess the effects of the minor, ancestral allele on PUFA and oxylipin concentrations, an increase in the study population is desirable.

## 5 Conclusion

In this pilot study in a routine clinical setting, we were able to confirm previous observations of significantly different blood fatty acid profiles depending on FADS1 genotype, with a lower D5D index in patients homozygous for the ancestral (minor) allele. Carriers of the derived genotype had a higher D5D index and higher AA concentrations and also showed a trend toward higher AA-derived thromboxane B2 and 12-HETE in blood plasma.

## Data availability statement

The original contributions presented in the study are included in the article, further inquiries can be directed to the corresponding authors.

## Ethics statement

The studies involving humans were approved by Ethics committee of Brandenburg Medical School. The studies were conducted in accordance with the local legislation and institutional requirements. The participants provided their written informed consent to participate in this study.

## Author contributions

MRa: Data curation, Methodology, Writing – review & editing, Investigation. ZW: Data curation, Visualization, Writing – original draft, Writing – review & editing. CL: Data curation, Writing – review & editing. JE: Data curation, Writing – review & editing, Conceptualization, Methodology, Supervision. MRo: Methodology, Writing – review & editing, Formal analysis, Investigation. AJ: Methodology, Writing – review & editing, Data curation. CS: Writing – review & editing. UE: Writing – review & editing. KW: Writing – review & editing, Conceptualization, Data curation, Methodology, Project administration, Supervision, Validation, Visualization, Writing – original draft. AP: Writing – review & editing, Investigation, Methodology, Project administration, Supervision.
